# Supplementary figures and images for: Synthesis, characterization and antibacterial activity of silver nanoparticles using Rhazya stricta
Source: PeerJ. 2018 Dec 17;6:e6086. doi: 10.7717/peerj.6086 (PMC6301278; doi:10.7717/peerj.6086)

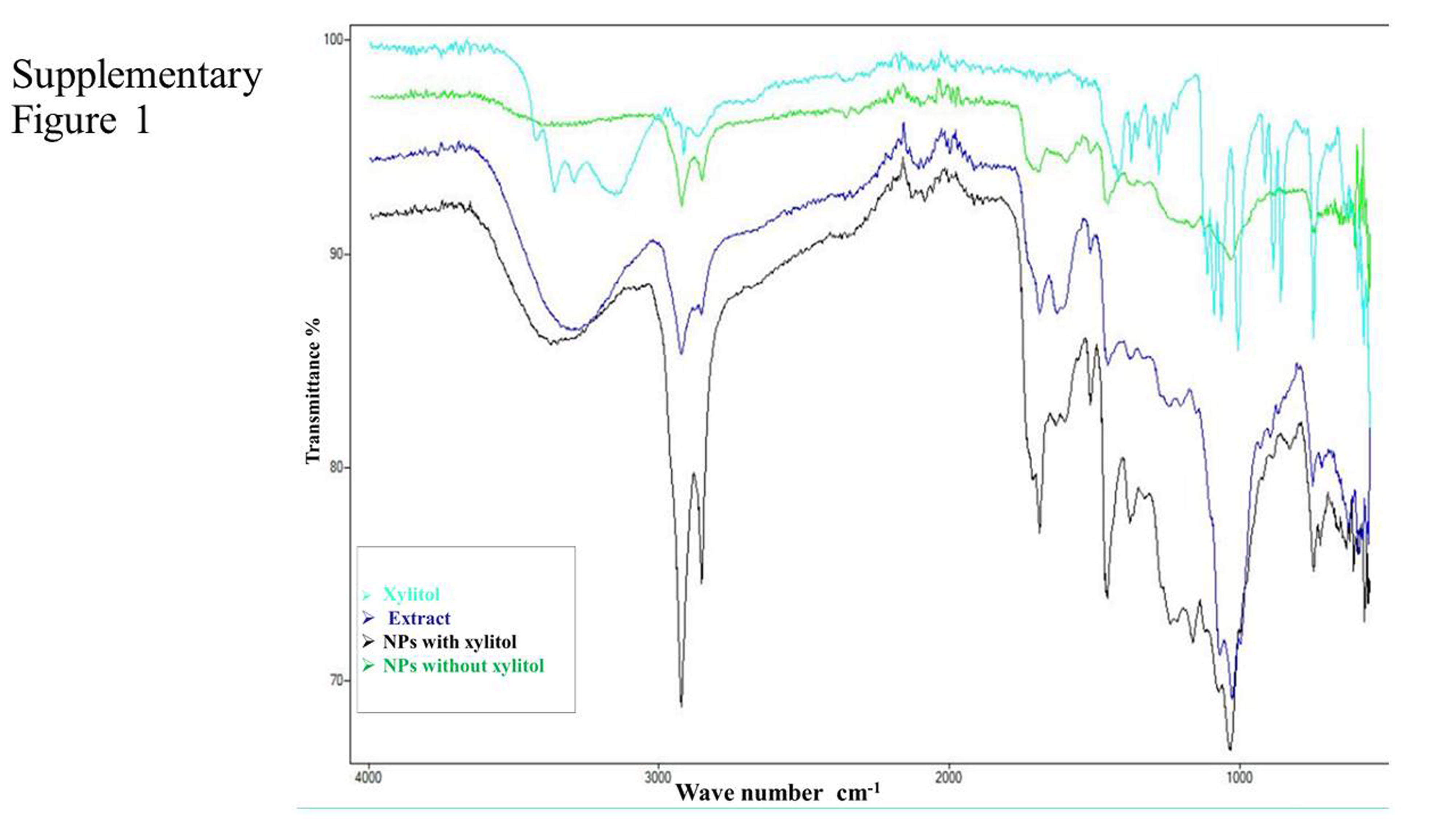

Supplement: Supplemental Information 5 [file peerj-06-6086-s005.jpg]
